# Understanding the early molecular changes associated with radiation therapy—A preliminary bulk RNA sequencing study

**DOI:** 10.1371/journal.pone.0316443

**Published:** 2025-03-03

**Authors:** Andrew Miller, Henning De May, David L. Rou, Jayant P. Agarwal, Sujee Jeyapalina

**Affiliations:** 1 Research, George E. Wahlen Department of Veteran Affairs Medical Center, Salt Lake City, Utah, United States of America; 2 Division of Plastic and Reconstructive Surgery, Department of Surgery, University of Utah, School of Medicine, Salt Lake City, Utah, United States of America; 3 Department of Biomedical Engineering, University of Utah School of Engineering, Salt Lake City, Utah, United States of America; 4 Undergraduate Research Opportunities Program, University of Utah, Salt Lake City, Utah, United States of America; 5 School of Biological Sciences, College of Science, University of Utah, Salt Lake City, Utah, United States of America; Huashan Hospital Fudan University, CHINA

## Abstract

**Introduction:**

Cancer is the second leading cause of death in the United States, with breast cancer being the most commonly diagnosed new cancer in women. Radiation therapy provides well-documented survival and recurrence benefits; however, it can lead to significant adverse effects, such as radiation-induced fibrosis (RIF), which can cause pain and result in poor aesthetic outcomes. The biological mechanisms underlying RIF are not entirely understood and require further investigation to identify potential intervention avenues. In this study, we investigated the biological response to radiation therapy by analyzing non-irradiated and irradiated tissues from breast cancer patients.

**Materials and methods:**

We collected tissue from breast cancer patients who underwent unilateral radiation and bilateral breast reconstruction. At the time of final reconstruction (post-radiation), samples were collected from both non-irradiated and irradiated reconstruction sites. These samples were analyzed using bulk RNA sequencing, histology, and immunohistochemistry (IHC).

**Results:**

In fibrous tissue capsules, CLCA2, COL4A5, and COL6A6 were differentially expressed and may be related to reduced micro-vascularization. CXCL9 and PTCHD4 were upregulated within the skin, possibly conferring an increased immune response, while multiple keratin-related genes (KRT6B, KRT17, KRT25, KRT28, and KRT75) were downregulated. In irradiated muscle tissue, there was increased expression of CXCL10 and downregulation of DCD. These results were confirmed using IHC.

**Conclusions:**

This study highlights the utility of bulk RNA sequencing studies in conjunction with IHC to identify target genes and biological processes responsible for RIF in tissues at final breast reconstruction. Due to the sample size limitation, further research is warranted to understand the role of keratin and collagen genes in regulating epidermal changes, vascularity, and fibrosis.

## Introduction

Breast cancer is the most common form of non-skin cancer in women in the United States, with an estimated 43,250 deaths in 2022 [1]. The standard of care for invasive breast cancer treatment is surgical tumor excision with adjuvant or neoadjuvant chemotherapy, with or without adjuvant radiation therapy [2]. While radiation therapy (RT) has significantly improved tumor-free survival [3,4], its use is associated with increased patient morbidity, including radiation-induced fibrosis (RIF), characterized by skin thickening and reduced compliance. Histologically, radiation-induced fibrotic tissue exhibits excessive collagen deposition, tissue atrophy, chronic inflammation, and fat necrosis [2,5,6]. At the cellular level, fibrosis involves fibroblast-to-myofibroblast trans-differentiation, increased extracellular matrix production, endothelial-to-mesenchymal transition, and excessive mesenchymal stem cell recruitment [7–9]. Biological factors influencing fibrosis include the volume of tissue treated, genetics, and epigenetics [5,10]. Treatment factors that increase the risk of developing RIF include the total dose of radiation therapy, the dose per fraction, and the course of treatment delivery.

Radiation can also directly damage DNA by causing double-stranded DNA breaks and crosslinking DNA chains or indirectly by forming free radicals such as reactive oxygen species (ROS) [11,12]. These molecular insults have been shown to result in RIF via a p16-associated pathway and CDK4/6-mediated cell cycle arrest and nucleo-shuttling of ataxia telangiectasia monomers (ATM) [13–15]. Moreover, radiation-induced cellular senescence has been demonstrated in multiple tissue types, including epithelial, endothelial, mesenchymal, and immune cells [16–22]. Supporting the role of cell senescence, Kim *et al*. demonstrated that radiation induces senescence-related gene expression profiles in endothelial cells [23]. Interestingly, some studies have shown that an increased risk of RIF is associated with a genetic variant in the ATM gene, which is responsible for repairing DNA double-strand breaks [24,25]. Other single-nucleotide polymorphisms have also been identified in genes encoding proteins, including superoxide dismutase 2. While molecular pathways have been proposed, a complete understanding of the mechanisms contributing to RIF remains limited, and no targeted treatment options currently exist.

This study aimed to investigate early molecular changes underlying the development of RIF in irradiated breast cancer patients. Tissue was obtained from patients who underwent unilateral breast irradiation followed by bilateral breast reconstruction. This allowed for paired analysis of non-irradiated and irradiated samples from the same patients using bulk mRNA sequencing, histology, and immunohistochemistry (IHC).

## Materials and methods

### Study design

**Fig 1:**
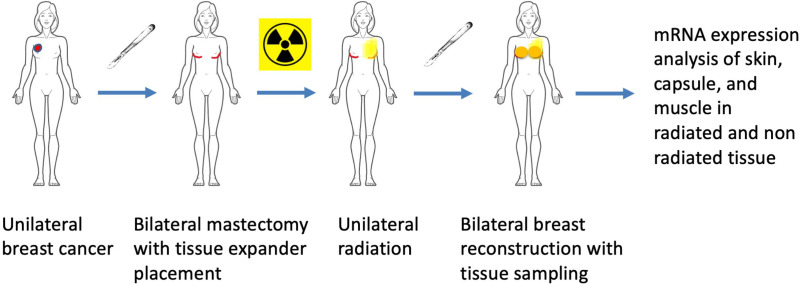
A flowchart showing the sequence of treatment and sample collection. Each patient enrolled in the current analysis underwent a bilateral mastectomy, followed by unilateral radiation of the affected breast. The paired tissue samples (non-irradiated and irradiated) were collected at the time of final breast reconstruction, which included skin, the pectoralis major muscle, and capsule tissue around the expander prostheses.

Using an institutionally approved protocol (University of Utah IRB #00047788), seven patients diagnosed with unilateral breast cancer were recruited for this Level II prospective observational pilot study. Written informed consent was obtained from each patient. Inclusion criteria included patients who elected to have a skin-sparing double mastectomy, received RT for a single breast, and underwent placement of tissue expanders under the pectoralis major muscle of both breasts with acellular dermal matrix. Patients were excluded based on smoking status, diagnosis of connective tissue disorders, diagnosis of autoimmune disorders, and the presence of diabetes. After completing RT, which lasted approximately seven weeks, patients underwent a second surgery to replace the tissue expanders with a smooth implant on the non-irradiated side and an autologous flap with or without a smooth implant on the irradiated side. More information on irradiation dose, location, timing, medications during treatment, and co-morbidities are provided in Table 1. During this surgery, tissue samples were collected from both reconstructed breasts (Fig 1). This unique study population enabled us to study the molecular side effects of radiation using a paired within-subject design between non-irradiated and irradiated tissue from each patient.

**Table 1:** Patient demographics and radiation treatment (RT) outcomes. Clinical fibrosis presence or absence was determined from a retrospective chart review conducted 7–8 years post-enrollment. Demographic data, RT details, medication use, and selected comorbidities were recorded both at enrollment and retrospective chart review.

Patient	Age at Mastectomy (Years)	Race/Ethnicity	Cancer Grade	Neo/Adjuvant Therapy	Irradiated Side	Irradiation Dose	Medications Used Between Irradiation Start and Reconstruction	Days from Irradiation to Reconstruction	Tobacco Use	Vascular or Autoimmune Diagnosis	Diabetes	Tissue Fibrosis (7–8 Years Post-Treatment)
1	60	White - Not Hispanic/Latino	Invasive ductal carcinoma: stage IIB (T2N1M0). ER(+)/PR(+)/ HER2(-)	Adjuvant dose dense paclitaxel, tamoxifen, letrozole	Left	3663 cGy to whole left breast, 3663 cGy to left axillary nodes, 1332 cGy mastectomy scar boost	Omega-3 FA, cholecalciferol	162	Never smoker	None	No	Normal skin, capsular contracture on the left
2	50	White - Not Hispanic/Latino	Invasive lobular carcinoma: stage IIB (T2N1M0). ER(+)/PR(+)/HER2(-)	Adjuvant Taxotere and cyclophosphamide, tamoxifen	Right	5000 whole right breast, 1000 mastectomy scar boost (5fract)	Omega-3 FA, cholecalciferol, loratadine, compazine, ibuprofen, ascorbic acid, calcium lactate, marijuana	126	Never smoker	None	No	Tightness of right breast
3	33	White - Not Hispanic/Latino	Infiltrating ductal carcinoma: stage IIB (T2 N1 M0) ER(+)/PR(+)/HER2(-) BRCA (+)	Neo-adjuvant, doxorubicin and cyclophosphamide followed by Taxol	Left	5000 cGy to whole left breast, 5000 cGy to left axillary lymph nodes breast, 1000 cGy mastectomy scar boost	Gabapentin, lorazepam and hydrocodone	117	Never smoker	None	No	Small/slight skin changes
4	33	Native American - Not Hispanic/Latino	Invasive ductal carcinoma of the left breast: stage IIA, (T1N1M0) grade 3, ER(+)/PR(+)/HER2(+)/BRCA(-)	Adjuvant doxorubicin, cyclophosphamide	Left	5000 cGy to whole left breast, 5000 cGy to left clavicular nodes, 1000 cGy mastectomy scar boost	Cephalexin, hydrocodone/acetaminophen, ibuprofen, lorazepam. ondansetron, compazine, senna.	173	Never smoker	None	No	None
5	51	White - Not Hispanic/Latino	Invasive ductal carcinoma: stage IIA (T1N1M0), ER(+)/PR(+)/HER2(-)/BRCA(-)	Tamoxifen	Right	5000 cGy to whole left breast, 4600 cGy right supraclavicular nodes followed, 1000 cGy mastectomy scar boost fx	Cholecalciferol, naproxen, omeprazole	134	Never smoker	None	No	None
6	67	White - Not Hispanic/Latino	Invasive ductal carcinoma: stage IIIa (T1N2M0), ER(-)/PR(-)/HER2(-)	Adjuvant doxorubicin, cyclophosphamide dose dense Taxol,	Right	4005 cGy to whole right breast, 4005 right sub clavicular nodes, 1000 cGy mastectomy scar boost fx	Aspirin, omeprazole, probiotics, Compazine, Ativan, Claritin, Tea Tree oral solution swish and spit, Zofran, Keflex	120	Never smoker	None	No	Small/slight skin changes
7	51	White - Not Hispanic/Latino	Invasive ductal cancer: stage IA, (T1N0M0). ER(+)/PR(+)/HER2(-)	Completed neoadjuvant dose dense doxorubicin, cyclophosphamide, dose dense Taxol	Right	5000 cGy R chest wall 25 Fx, 5000 cGy R sub clavicular nodes 25 Fx, No scar boost	Naproxen, omeprazole, dexamethasone, lysine, loratadine, lorazepam, ondansetron, olanzapine, trazadone	96	Never smoker	None	No	Small/slight skin changes

### Sample collection

During the removal of tissue expanders and placement of silicone implants and/or autologous tissue, three different tissue types were collected from non-irradiated and irradiated breasts. Skin samples were taken centrally from the mastectomy scar. Muscle samples were obtained from the underlying pectoralis major muscle. If muscle was not found directly under the mastectomy scar, it was taken from a point superior to the incision within the radiation field. A capsule sample was taken from around the expander directly inferior to the mastectomy scar (Fig 1). In all cases, samples were taken from the radiated field and the corresponding location in the contralateral breast. Tissue samples were immediately submerged in RNAlater™ (Sigma-Aldrich, St. Louis, MO, USA), stored at 4°C overnight, and subsequently stored at −80°C until further use. Additional fresh tissue samples were frozen and stored at − 80°C for histopathological analyses.

### Total RNA extraction, sequencing, and analyses

Total RNA was extracted from samples processed in RNAlater using RNA extraction kits (Qiagen AllPrep Universal DNA/RNA/miRNA Kit, Qiagen, Venlo, Netherlands, Germany). RNA Integrity Number (RIN) values were recorded using electrophoresis (Agilent Technologies RNA ScreenTape Assay, Agilent, Santa Clara, CA, USA). Extracted RNA underwent ribosomal depletion and library preparation (Illumina TruSeq Stranded RNA Kit with Ribo-Zero Gold, Illumina, San Diego, CA, USA). Single-end 50 bp sequencing was performed by the High-Throughput Genomics and Bioinformatics Analysis Shared Resource at the Huntsman Cancer Institute, University of Utah. Sequence quality was assessed using MULTIQC [26], incorporating RNA-seq metrics from FASTQC v0.11.5 [27], SAMTOOLS v1.8, and PICARD v2.9.0. Sequences were then aligned to the reference genome (GRCh38) using STAR v2.7.3a [28–30]. A variance stabilization transformation was applied to the data using the vst function from DESeq2 v1.32.0 [31] before performing the principal component analysis (PCA). Genes were annotated using Ensembl v104. For each tissue type (skin, muscle, and fibrous capsule), non-irradiated samples were compared to irradiated samples using DESeq2 to identify differentially expressed genes (DEGs). Heat maps of the log-normalized counts of DEGs from each tissue type were created using the heatmap package [32]. DEGs from each differential expression analysis were used by topGO [33] to perform the enrichment analysis on Gene Ontology (GO) Biological Process (BP) terms [34]. The sequencing data has been deposited in the Gene Expression Omnibus (GEO) repository under accession number GSE278183.

### Histological staining

Frozen tissues were thawed and fixed in 10% formaldehyde (Sigma-Aldrich). After embedding in paraffin, 5 μm thick cross-sections were cut in the coronal plane using a microtome. The sections were mounted on glass slides and stained with H&E and Masson’s trichrome stains following clinically validated protocols. Light microscopy (Eclipse Ni-U, Nikon Corporation, Tokyo, Japan) was employed to evaluate the tissue samples for morphology, fiber orientation, and cellular density.

### Immunohistochemical staining (IHC)

Fresh frozen samples from each tissue type were embedded in an optimized cutting medium (TISSUE-TEK O.C.T, Sakura Finetek, Torrance, CA, USA), and 10 μm tissue sections were obtained using a cryostat (Shandon Cryotome E, Thermo Shandon, Cambridge, UK). The tissue sections were mounted on glass slides (Diamond White Glass, Globe Scientific, Mahwah, NJ, USA) and stored at − 20°C until further use. For IHC staining, frozen tissue samples were removed from the freezer and allowed to equilibrate at room temperature for 15 minutes to dry. Next, the samples were fixed with 100% acetone (Sigma-Aldrich, St. Louis, MO, USA) for 15 minutes. The fixed tissues were then washed with 1x phosphate-buffered saline (PBS) three times for five minutes each and permeabilized with 0.3% Triton X-100 in 1x PBS (Sigma-Aldrich) for one hour, except for samples with COL6A6, CLCA2, CXCL9, CXCL10, and TMEM255A, as these proteins are generally located on the plasma membrane or secreted. Therefore, permeabilization was not required for these samples. After minimizing non-specific binding with a blocking solution (BlockAid Blocking Solution, Thermo Fisher Scientific, Waltham, MA, USA) for one hour, 10 μl of the respective primary antibodies (Prestige Antibodies: HPA000453, HPA061168, HPA047192, HPA045239, SAB4301841, HPA048470; Sigma-Aldrich) and 11H1L14 (Invitrogen, Frederick, MD, USA) were applied to the samples at the following dilution ratios: 1:200 for HPA000453, HPA061168, HPA045239, and HPA048470; 1:50 for SAB4301841; 1:500 for HPA047192; and 1:100 for 11H1L14. The samples were then incubated at 4°C overnight. Subsequently, the tissues were washed with 1x PBS three times for five minutes each, incubated with the respective secondary antibodies (ab96886, Abcam, Cambridge, UK) diluted 1:1000 for one hour, and washed again with 1x PBS three times for five minutes each. After washing, 4′,6-diamidino-2-phenylindole (DAPI) mounting medium (Abcam) and antifade (ProLong Gold, Thermo Fisher Scientific) were applied. The cover-slipped samples were imaged using a confocal laser microscope (FV1000, Olympus Corporation, Tokyo, Japan).

### Vascular density measurements

Collagen IV IHC images taken at 40x magnification were imported into ImageJ and converted to grayscale. The images were then processed using auto-threshold with the minimum setting and subsequently skeletonized. Sixteen regions were randomly selected across the image to measure vascular density using the vessel analysis plugin. The computed values are reported as the vessel area within 44.1 mm^2^.

### Retrospective chart review

Patient chart reviews were conducted in December 2022. At the time of the chart review, all patients in the study were 7 to 8 years post-enrollment and were cancer survivors. Information was gathered on each patient’s irradiation dose, treatment timeline, clinical outcomes (including skin changes and capsular contracture), and medications taken during the study period (from diagnosis to reconstruction). The charts were also reviewed for pre-cancer and post-treatment diagnoses of comorbidities such as smoking, vascular disorders, diabetes, and medications that may affect tissue fibrosis.

### Statistical analyses

DESeq2 v1.32.0 [31] was used to perform the differential expression analyses. This tool normalizes and transforms the expression data before fitting a generalized linear model based on a negative binomial distribution. RIN and irradiation status were included as covariates, and the Benjamini-Hochberg multiple testing correction method was applied to the resulting p-values with a false discovery rate (FDR) < 0.05. Significant genes identified from the differential expression analyses were used for enrichment analyses. Due to the overlap of genes between BP terms from GO and the assumption of independence between terms, multiple testing correction was not applied. Instead, the topGO elim algorithm was used. When a node is found to be significant using the elim algorithm, the corresponding genes were marked and removed from all parent nodes before a Fisher’s Exact test was performed on the next parent node. Based on a retrospective chart review, an additional differential expression analysis was performed for irradiated samples, comparing patients who did not develop RIF to those who did. Unless otherwise stated, paired t-tests were performed for all other analyses using Stata 15.0 (StataCorp LLC, TX, USA).

## Results

One sample was removed after RNA sequencing due to poor sequence alignment (17% uniquely mapped reads, patient #4 irradiated skin), and genes with fewer than ten total reads were excluded from further analyses. Batch effects were not observed in the PCA visualization (data not shown); however, two samples were contained in alternate clusters (patient #3 irradiated muscle and patient #3 irradiated skin). These samples were likely mislabeled but were removed from further analysis as this could not be verified.

**Fig 2:**
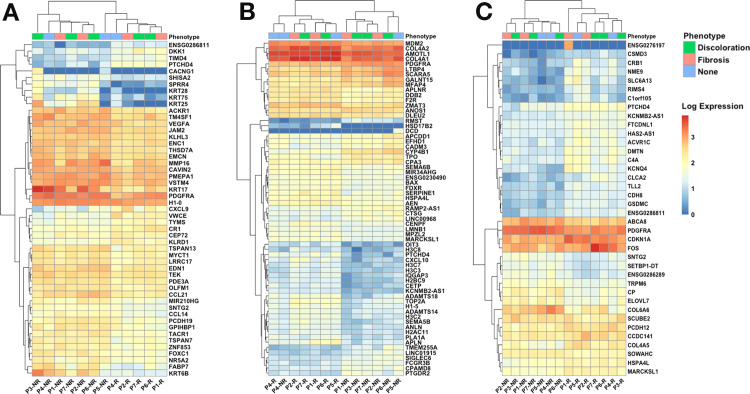
A set of representative heatmaps showing the normalized log-expression values for differentially expressed genes (DEGs). (A) Fifty-one DEGs were identified from skin samples. (B) Sixty-five DEGs were identified from muscle samples. (C) Thirty-nine DEGs were identified from the fibrous capsule. The top row above the heatmap indicates the phenotypic traits experienced by patients: capsular contracture (fibrosis), skin changes (discoloration), or none. Abbreviations: Patient (P); Non-Irradiated (NR); Irradiated (R).

In total, thirty-nine samples were included for RNA sequencing analysis after applying quality control metrics. On average, sequencing samples had a coverage of 24 million reads and a RIN of 7.52. Guanidine-cytosine (GC) content ranged from 44% to 61%, and ribosomal transcripts were effectively depleted, with less than 0.1% of mapped reads consisting of ribosomal reads. For skin, muscle, and fibrous tissue samples, 31,424, 25,834, and 28,136 genes were included in the differential expression analyses, respectively. For each tissue type, the samples generally clustered by irradiation status (Fig 2A–2C).

**Fig 3:**
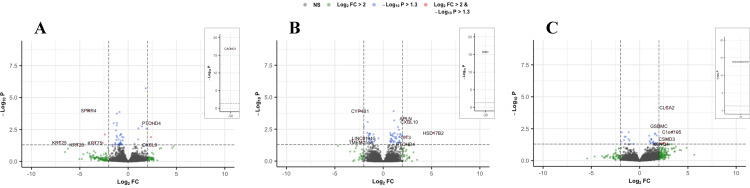
A set of volcano plots by tissue type. Insets show genes with exceptionally low or high log fold-change values. (A) Skin samples. (B) Muscle samples. (C) Fibrous capsule samples. Vertical dotted lines correspond to a log-fold change (FC) of ± 2. Horizontal dotted lines indicate a p-value of 0.05.

In irradiated skin samples, 51 significant DEGs were identified (Fig 3A), with CACNG1 being the most downregulated gene (~30-fold; Fig 3A inset). SPRR4 and FABP7 were also downregulated, along with multiple keratin genes (KRT6B, KRT17, KRT25, KRT28, and KRT75). PTCHD4 and CXCL9 were the most upregulated genes in the skin. Significant GO BP terms associated with these keratin genes include ‘intermediate filament organization’ (KRT17, KRT25), ‘hair follicle morphogenesis’ (KRT17, KRT25), and ‘keratinization’ (KRT17, SPRR4), while CXCL9 is involved in local inflammatory responses.

In irradiated muscle tissue, 65 significant genes were identified (Fig 3B). Notably, DCD was approximately 30-fold downregulated. RMST, CYP4B1, TMEM255A, and LINC01915 were also downregulated. Additional highly upregulated genes included HSD17B2, CXCL10, OIT3, APLN, and PTCHD4. Associated BP terms identified with these genes include ‘angiogenesis’ (APLN, CXCL10) and ‘positive regulation of cell migration’ (CXCL10).

In irradiated capsular tissue, 39 DEGs were identified (Fig 3C). The most upregulated genes included SLC6A13, C1orf105, CLCA2, CSMD3, RIMS4, NME9, CRB1, KCNQ4, and GSDMC. Significant BP terms in the fibrous tissue included ‘positive regulation of cell death,’ (SLC6A13) ‘supramolecular fiber organization,’ (TLL2) and ‘response to radiation’ (CRB1).

A differential expression analysis comparing irradiated samples from only patients who did not experience RIF with those who did revealed 8 genes for skin, 10 genes for muscle, and 9 genes for capsular tissue. In skin, EFEMP1 was downregulated while FER1L6, PPM1E, COL22A1, NEFM, GBP6 were upregulated. In muscle, LYPD6B, SERPINB4, KRT15 were downregulated while PSAPL1, CDSN, and ESRP1 were upregulated. In capsular tissue, SIK1, AWAT1, and TBC1D3B were downregulated with no protein-coding genes upregulated.

**Fig 4:**
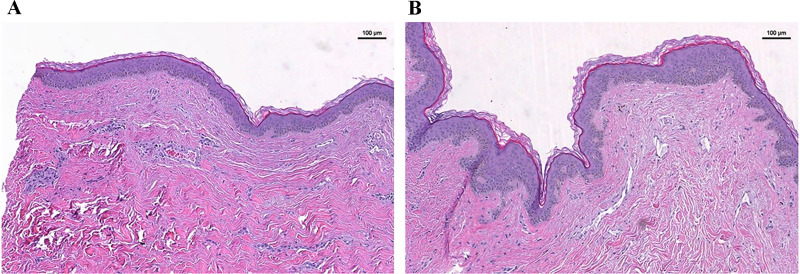
A representative set of H&E stained skin tissue samples. (A) Non-Irradiated. (B) Irradiated. Irradiated skin samples experienced increased epidermal thickness compared to non-irradiated samples. Scale bar = 100 mm.

As stated in the Introduction, epithelial thickening is a known outcome of RT. Therefore, in order to quantify the epithelial thickness, the H&E stained samples were used. Using highly magnified light microscopic images, thickness of the epidermis along the skin was recorded and compared between the non-irradiated and irradiated skin sites (Fig 4). Although generally non-irradiated tissue had a thinner epidermis (50.5 ± 9.2 μm) compared to irradiated tissue (61.0 ± 7.4 μm), there was no statistically significant difference in epidermal thickness between skin samples based on irradiation status (p = 0.065).

**Fig 5:**
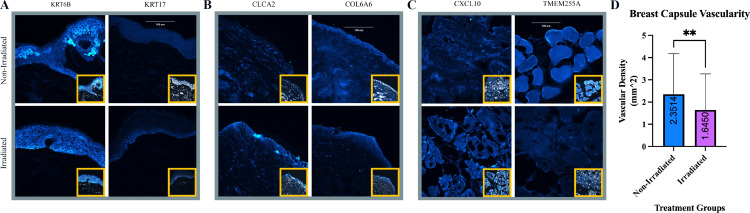
Confocal images of selected antibodies and vascular density bar chart. Representative confocal images of (A) skin, (B) capsule, (C) muscle samples stained for selected antibodies corresponding with up- and down-regulated genes. The inset shows the corresponding DAPI-stained sample. Within the irradiated skin tissue, a reduced translation of KRT6B and slightly increased translation of KRT17 were noted. While irradiated capsules showed increased expression of CLCA2, irradiated muscle demonstrated increased translation of CXCL10 and reduced translation of TMEM225A. (D) Bar chart of quantified collagen IV expression levels in non-irradiated and irradiated capsule tissue (p < 0.01).

**Fig 6:**
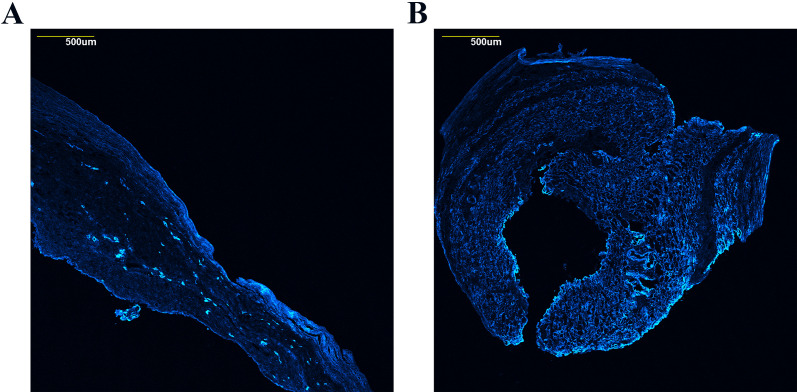
Confocal images of COL4A1 expression in capsular tissue. Representative confocal images showing COL4A1 expressions (representing endothelial or epithelial basement membrane, a surrogate for angiogenesis) in (A) non-irradiated and (B) irradiated breast capsules.

To confirm that over- or under-expression did indeed result in a corresponding trend in translated protein, we picked representative genes for confirmation. From the data, seven DEGs with the most significant changes were selected to confirm the translation of gene expression levels using IHC techniques (Fig 5). The data showed that, within the skin, KRT6B and KRT17 transcription were downregulated in the irradiated tissue. At the same time, CXCL9 was significantly upregulated in the epidermis. Notably, although overall KRT17 transcription was reduced, irradiation treatment induced the translation of K17 within the basal cell layer. Increased translation of CLCA2 was observed in the irradiated capsule, while COL6A6 expression was downregulated, possibly indicating changes in vascularization. Vascular densities were therefore computed for the unit area using the capsular IHC images, which was confirmed with COL4A1 (Fig 6). The results demonstrated a statistically significant reduction in vascularity in irradiated tissues (p < 0.01), with 2.315 ± 1.826 vessel area/mm^2^ in non-irradiated tissues compared to 1.645 ± 1.622 vessel area/mm^2^ (Fig 5D). In irradiated muscle, CXCL10 was upregulated while TMEM255A was downregulated, suggesting local inflammation and immune cell recruitment.

At the time of this manuscript preparation, a retrospective chart review was conducted. The patient demographic characteristics and the clinical presentation of the fibrosis are shown in Table 1. On average, patients were 49.8 ± 12.8 years old at diagnosis. Six patients (86%) were identified as White, and one (14%) was a Native American. The average time from the end of irradiation to final breast reconstruction was 133 ± 27 days. Based on the retrospective chart review, seven to eight years post-treatment, only two patients (28.6%; Patients 1 and 2) developed signs of capsular contracture on the irradiated side, while three patients (42.8%; Patients 3, 6, and 7) showed skin discoloration. At the time of surgery or chart review, none of the patients were diagnosed with vascular disorders, diabetes, tobacco exposure, or any known exposure to agents proposed to reduce radiation-induced fibrosis, such as pentoxifylline, hyperbaric oxygen, or vitamin E (Table 1). All patients were alive and cancer-free at the time of the chart review (December 2022).

## Discussion

This study was designed to enhance our understanding of the early molecular changes associated with RT. To achieve this, we compared the molecular profiles of non-irradiated versus irradiated tissues at final breast reconstruction using bulk RNA sequencing. This approach allowed for a robust analysis of DEGs in three tissue types: skin, muscle, and capsule. The data were then confirmed using IHC staining of these three tissue types, demonstrating the spatial expression of respective proteins.

Literature reports that a substantial proportion of radiation-related side effects involve changes within the dermis and epidermis, including epithelial thickening, hair loss, and reduced wound healing [35,36]. Through bulk RNA sequencing of the skin, we identified keratin-family genes, particularly KRT6B, KRT17, KRT25, KRT28, and KRT75. Keratins are primary components of hair and epidermis and play essential roles in tissue structure, integrity, and function [37]. Substantial evidence shows that the expression of K6 paired with K16 or K17 is crucial for wound healing, and aberrant expression can lead to psoriasis and increased skin thickness [38]. We observed reduced expression of KRT6 with radiation, but in murine and *in vitro* studies, it is linked to increased epithelialization and epithelial fragility [39]. Our IHC data suggest that the effect of radiation is more complex, as there was an increased translation of KRT17 in the basement membrane despite the global downregulation of its RNA transcript. These topographical changes in expression warrant further investigation. The under-expression of these keratin genes in irradiated tissue may partly explain radiation-induced epidermal thickening and reduced wound healing capabilities.

We observed significant changes in the immune microenvironment of irradiated tissue. Notably, there was upregulation of CXCL9 in the skin and CXCL10 in the muscle. Both are interferon-induced chemokines that act as ligands for CXCR3, regulating local immune responses and fibroblast migration [40]. Additionally, both ligands have been implicated in pathological fibrosis following tissue damage [41–43]. IHC imaging of CXCL9 translation (Fig 5) shows prominent expression within the epidermis, highlighting its potential role in mediating inflammation. The superficial expression pattern of this gene in the epidermis suggests the potential for topical therapeutic approaches to suppress this gene could be considered as a possible target for preventing the effects of radiation on skin tissue.

The antimicrobial gene dermcidin (DCD) was 30-fold downregulated in irradiated muscle. DCD is linked to cell survival and breast cancer cell migration through Wnt signaling [44–47]. While DCD expression is associated with disease progression and survival in breast cancer patients, its role after irradiation treatment is not well understood [46]. Other notable genes in irradiated muscle tissue include HSD17B2, APLN, OIT3, and PTCHD4. The implications of these genes for capsular contractures have yet to be discovered.

Concerning the breast prosthesis capsule, we observed changes in multiple collagen genes. Notably, there was downregulation of COL4A5 (collagen type IV alpha 5 chain) and upregulation of COL4A6 (collagen type IV alpha 6 chain). Both genes encode subunits of collagen type IV, a major component of basement membranes, including those in the endothelium and epithelium. Based on the known effects of radiation on small vasculature, these changes may represent a reduction in capsular vascularization, which was confirmed by IHC staining. Further research is needed to determine whether changes in collagen gene expression are driving the decrease in capsular vascularization, which needs controlled animal studies to investigate the effect of radiation.

This paper identified trends but no significant differences in changes to epidermal thickness. We believe this lack of significance may be attributed to the time point of RNA analysis and histology following the completion of irradiation treatment (133 ± 27 days). This time point was chosen to analyze early molecular changes in tissues at final breast reconstruction, occurring before most long-term side effects of radiation become evident (typically four months to a year) (5).

Our retrospective chart review revealed that two of the seven women (~28.6%) developed unilateral capsular contracture in their irradiated breasts. This finding is consistent with the literature, which reports a 10–25% incidence rate for capsular contracture [48,49]. Notably, one of the two patients who developed unilateral fibrosis had received a lower dose of radiation due to toxicity concerns during treatment. It is well established that higher irradiation doses significantly increase the risk of fibrosis [50]. Larger daily doses are associated with more complications, including fibrosis, with incidence rates reaching as high as 75–100% in some studies [51]. The development of fibrosis in this patient, despite receiving lower doses, suggests that other underlying genetic or environmental factors may have increased their risk of RIF. Furthermore, multiple comorbidities such as diabetes, scleroderma, rheumatoid arthritis, systemic lupus erythematosus, and smoking are known to elevate the risk of RIF [52–54]. In our cohort, these comorbidities and medications known to reduce fibrosis—such as montelukast, pentoxifylline, hyperbaric oxygen, or vitamin E—were not noted during the retrospective chart review and are unlikely to have contributed to RIF outcomes in this study. These observations suggest radiation was the most likely underlying factor driving molecular and clinical changes in irradiated breast tissue.

This study is based on seven patients, with five to seven samples per tissue type. It is, therefore, limited by its small sample size and the genetic background of the patients. While our rate of capsular contracture (28.6%) aligns with the literature, this finding was based on only two patients, making it difficult to perform a robust statistical analysis between these patients and the rest of the cohort. Additionally, the timing of the second stage of reconstruction was not controlled, which may have affected the identification of DEGs. Nevertheless, this study demonstrates the potential of new and emerging RNA techniques and suggests the need for controlled studies to better understand the mechanisms of RIF. Lastly, samples were collected at a single time point after RT, ranging from 96 to 173 days between subjects.

In conclusion, a within-subjects analysis between non-irradiated and irradiated tissue in breast cancer patients was performed investigating the molecular effects of RT. Molecular changes were observed at both the transcript and protein level following RT in skin, muscle, and capsular tissues utilizing RNA sequencing, histology and IHC. Further targeted RNA sequencing studies using murine models and/or alternative sequencing technology–such as spatial transcriptomics– will provide a deeper understanding of the molecular effects of radiation on the reconstructed breast tissues, which is currently being undertaken by our group.
